# Evaluation of pemetrexed and etoposide as therapeutic regimens for human papillomavirus-positive oral and oropharyngeal cancer

**DOI:** 10.1371/journal.pone.0200509

**Published:** 2018-07-11

**Authors:** Yi Rang Kim, Bada Lee, Mi Ran Byun, Jong Kil Lee, Jin Woo Choi

**Affiliations:** 1 Department of Hemato-Oncology, Yuseong Sun Hospital, Daejeon, Republic of Korea; 2 Department of Pharmacology, College of Pharmacy, Kyung Hee University, Seoul, Republic of Korea; 3 Department of Life and Nanopharmaceutical Sciences, Kyung Hee University, Seoul, Republic of Korea; Queen Mary University of London, UNITED KINGDOM

## Abstract

Although human papillomavirus (HPV) positive oral and oropharyngeal cancers have distinct epidemiologic and molecular characteristics compared to HPV-negative cancers, all patients with oral and oropharyngeal cancers received same standard regimen regardless of HPV status. For these reasons, specific regimens for patients with HPV-positive oral and oropharyngeal cancer are needed. Differentially expressed genes (DEG) between HPV-positive and HPV-negative oropharyngeal cancers were re-analyzed and categorized from public database. Then, druggable targets to HPV-positive oral and oropharyngeal cancer were identified and were validated with E6/E7, which is oncogene of HPV, transfected oral and oropharyngeal cancer cell lines and HPV infected cell lines. In DEG analysis, HPV-positive oral and oropharyngeal cancer showed distinct disease entity from HPV-negative cancers. Unlike HPV-negative oral and oropharyngeal cancer, thymidylate synthase (TS) and topoisomerase II (Topo II) were overexpressed in HPV-positive cancers. Transfection of Lenti-virus containing E6/ E7 to HPV-negative oral and oropharyngeal cancer cells induced upregulation of TS and Topo II in those cells. Although cisplatin, which is standard regimen in head and neck cancers, showed more effectiveness in HPV-negative cells, 5-FU and pemetrexed, which are TS inhibitors, or etoposide, which is Topo II inhibitors, worked more effectively in HPV-positive cells. In addition, cisplatin/etoposide and cisplatin/pemetrexed combination regimens showed synergic effects in HPV-positive cells. Pemetrexed or etoposide alone, or in combination with other chemotherapeutic agents such as cisplatin, can be used as novel substitutes in a regimen of concurrent chemoradiotherapy or a palliative regimen for HPV-positive oral and oropharyngeal cancer patients. However, a well-designed clinical trial is needed.

## Introduction

Worldwide, more than 550,000 cases of head and neck cancer are newly diagnosed each year and approximately 380,000 deaths are attributed to the disease.[1] In the United States, head and neck cancer accounts for three percent of total malignancies.[2] Tobacco, alcohol, and viral infections, such as human papillomavirus (HPV) and Epstein-Barr virus (EBV), are well-known risk factors for head and neck cancers.[3–5] However, since the late 1980s, non-oropharyngeal cancers, such as laryngeal, hypopharyngeal, and oral cavity cancers have decreased owing to the decrease in smoking rates.[6] In contrast, the incidence of oropharyngeal cancer has increased [7] and 50–80% of cases were attributable to HPV; the dramatic rise in the incidence of oropharyngeal cancers and HPV, which can be transmitted through sexual contact and oral-genital contact, are closely linked.[8–10] Among HPVs, HPV-16 is well known carcinogenic phenotype. Unlike the low prevalence of HPV-16 in oral cavity cancers (14.3%) and laryngeal cancers (13.4%), the high prevalence of HPV-16 in oropharyngeal cancers (40.6%) is also connected to the increased incidence of oropharyngeal cancers.[11]

In comparison with HPV-negative oropharyngeal cancer, 59 differently expressed genes have been already identified in HPV-positive oropharyngeal cancer, so it is predicted that HPV-positive oropharyngeal cancers have distinct epidemiologic, pathologic, and molecular characteristics.[10, 12, 13] Consequently, it is inevitable that HPV-positive oropharyngeal cancers will have different radiosensitivities and chemosensitivities to specific chemotherapeutic drugs. Despite the clear evidence from continuous studies, which suggest that HPV-positive oropharyngeal cancer forms an independent disease entity, all cases of oropharyngeal cancer have been managed independent of HPV status. Although O’Sullivan et al. announced that, unlike patients with HPV-negative oropharyngeal cancer, the overall survival of patients with HPV-positive oropharyngeal cancer was not correlated with UICC/AJCC 2010 TNM stage, patients with HPV-associated head and neck cancers were treated with the same standard regimen as for HPV-negative head and neck cancers.[9, 14–16] In addition, although several epidemiologic studies on the incidence of HPV in head and neck cancers or prognostic studies between HPV-positive and -negative head and neck cancers have been performed, no studies have been conducted to separate the chemotherapy regimen between the HPV-positive and -negative cancers.[17–20] For these reasons, the consensus that separate clinical trials are needed for HPV-related and -unrelated head and neck cancers has begun to emerge; several clinical trials (NCT01855451, NCT01898494, NRG HN-002) are now in progress.[6, 16]

In this article, to meet the needs for separate treatments based on HPV status, we identified the differently expressed genes between HPV-positive and HPV-negative oral and oropharyngeal cancers, determined drugs that specifically targeted the overexpressed genes, and selected candidate drugs for a novel regimen of HPV-positive oral and oropharyngeal cancers.

## Materials and methods

### Cell line and chemicals

YD10B cells and Ho-1-N-1 were provided by Korean Cell Line Bank and Japan Cell Line Bank, respectively, and cultured in Dulbecco’s modified Eagle’s medium (DMEM; Hyclone) supplemented with 10% fetal bovine serum (FBS) and 2% penicillin/streptomycin. HNSCC (human Head and Neck Squamous Cell Carcinoma) cell lines (HPV negative cell line, UM-SCC-1 and HPV positive cell line, 93-VU-147T) were a gift from Dr. Jong-Lyel Roh (Ulsan University, Seoul, Korea). UM-SCC-1 and 93-VU-147T cells were maintained in DMEM containing 10% FBS, 100 units/ml penicillin, and 100 μg/ml streptomycin. Hela, SiHa and SKOV3 are provided from Korean Cell Line Bank and cultured in Roswell Park Memorial Institute medium (RPMI) with 10% FBS and 2% penicillin/streptomycin. Cisplatin, paclitaxel, pemetrexed, 5-FU, and etoposide were purchased from Sigma Aldrich (U.S.) and dissolved in DMSO for treatment to cells. The DNA vectors for E6 and E7 were obtained from Addgene (U.S.).

### Reverse transcription (RT)-PCR

Total cellular RNA was extracted from each cell by using a RNeasy plus mini kit (Qiagen) and the complementary DNA product was synthesized by using Transcriptor First Strand cDNA Synthesis Kit (Roche Applied Science, Mannheim, Germany) in accordance with the manufacturer’s instructions. The following primers for PCR were used: HPV-16 E6, Forward (F) 5'-ATGCACCAAAAGAGAACTGC-3', Reverse (R) 5'-TTACAGCTGGGTTTCTCTAC-3'; HPV-16 E7, (F) 5'-GTAACCTTTTGTTGCAAGTGTGACT-3', (R) 5'-GATTATGGTTTCTGAGAACAGATGG-3'; thymidylate synthase (F) 5'-TTACCTGAATCACATCGAGC-3', (R) 5'-ATATCCTTCGAGCTCCTTTG-3'; topoisomerase II: (F) 5'-TGCCTGTTTAGTCGCTTTC-3', (R) 5'-TGAGGTGGTCTTAAGAAT-3; and GAPDH: (F) 5'-TGAAGGTCGGAGTCAACGGATTTGGT-3', (R) 5'- ATGTGGGCCATGAGGTCCACCAC-3'. cDNA was amplified by using the Accu Power Hot Start PCR Pre Mix (Bioneer, Daejeon, South Korea) with the following conditions: 30 cycles of 30 s at 94 °C, 30 s at 60 °C, and 40 s at 72 °C. The amplified products were then separated on a 1.0% agarose gel, stained with 0.1 μg/mL ethidium bromide, and photographed under UV light (Bio-Rad, Hercules, CA, USA)

### Gene expression analysis

Original gene expression data was obtained from Martinez et al. [21] Based on the data set, we calculated differentially expressed gene (DEGs) with a basis of p = 0.01 and FC<2. A linear mixed model was used to examine variability in gene expression by HPV infection. We fitted the mixed-effects model in R with the lmer function in the lme4 package. The p-values to assess the significance of the age effect were calculated from the chi-square distribution with one degree of freedom using the likelihood ratio as the test statistic. The p-values adjusted for multiple testing were computed by controlling the false discovery rate (FDR) with the Benjamini-Hochberg procedure in R and using a threshold of 0.01.

### Protein-protein interaction data

The DEG dataset was inserted as an input list for Cytoscape 2.0 and KEGG and STRING were called as template data.

### MTT assay

The cell viability was measured by using an MTT assay (Promega Ltd) in accordance with the manufacturer’s protocol. Briefly, 5 × 10^3^ cells/well were seeded in 96-well plates. After treatment with the test chemicals in the presence or absence of hydroxyurea pre-treatment (10 μmol/mL), the cells were incubated with 5 mg/mL MTT for 4 h. The medium was subsequently removed; 150 μL solubilization solution and 150 μL stop solution were added and incubated at 37 °C for 4 h. The absorbance of the reaction solution was measured at 570 nm. The cell growth inhibition was calculated from the following equation: (1 –absorbance of experimental group/absorbance of control group) × 100%.

### LDH assay

The cells were seeded into 24-well plates at 5 × 10^4^ cells/well and treated with each of the test chemicals for 2 days. The media were prepared separately. The LDH Cytotoxicity Assay kit (Cayman Chemical Company; Ann Arbor, MI, USA) was used as described by the manufacturer. Briefly, the cells were grown at 37 °C and 5% CO_2_ in DMEM supplemented with 10% heat-inactivated FBS and 1% penicillin/streptomycin and seeded at 2 × 10^4^ cells/well in 96-well plates. After 48 h, 100 μL of the supernatant of the cultured cells was transferred from each well to the corresponding wells of a new plate and 100 μL of reaction solution was added to each well. The plates were incubated with gentle shaking in an orbital shaker for 30 min at 18–21 °C (room temperature) and the absorbance of each well at 490 nm was measured by using a plate reader.

### TUNEL assay

UM-SCC-1 and 93-VU-147T cells were seeded on cover slip and treated with drug or vehicle for 24 h. Then, the cells were fixed with 4% paraformaldehyde for 1 h at room temperature and permeablized with 0.1% Triton X-100 in PBS for 10 min at 4 °C. Cell death was stained by TUNEL assay kit (*In Situ* Cell Death Detection Kit, TMR red, Roche) and analyzed by a confocal microscopy.

## Results

### HPV-positive oral cancer is a distinct disease entity from HPV-negative oral cancer

**Fig 1:**
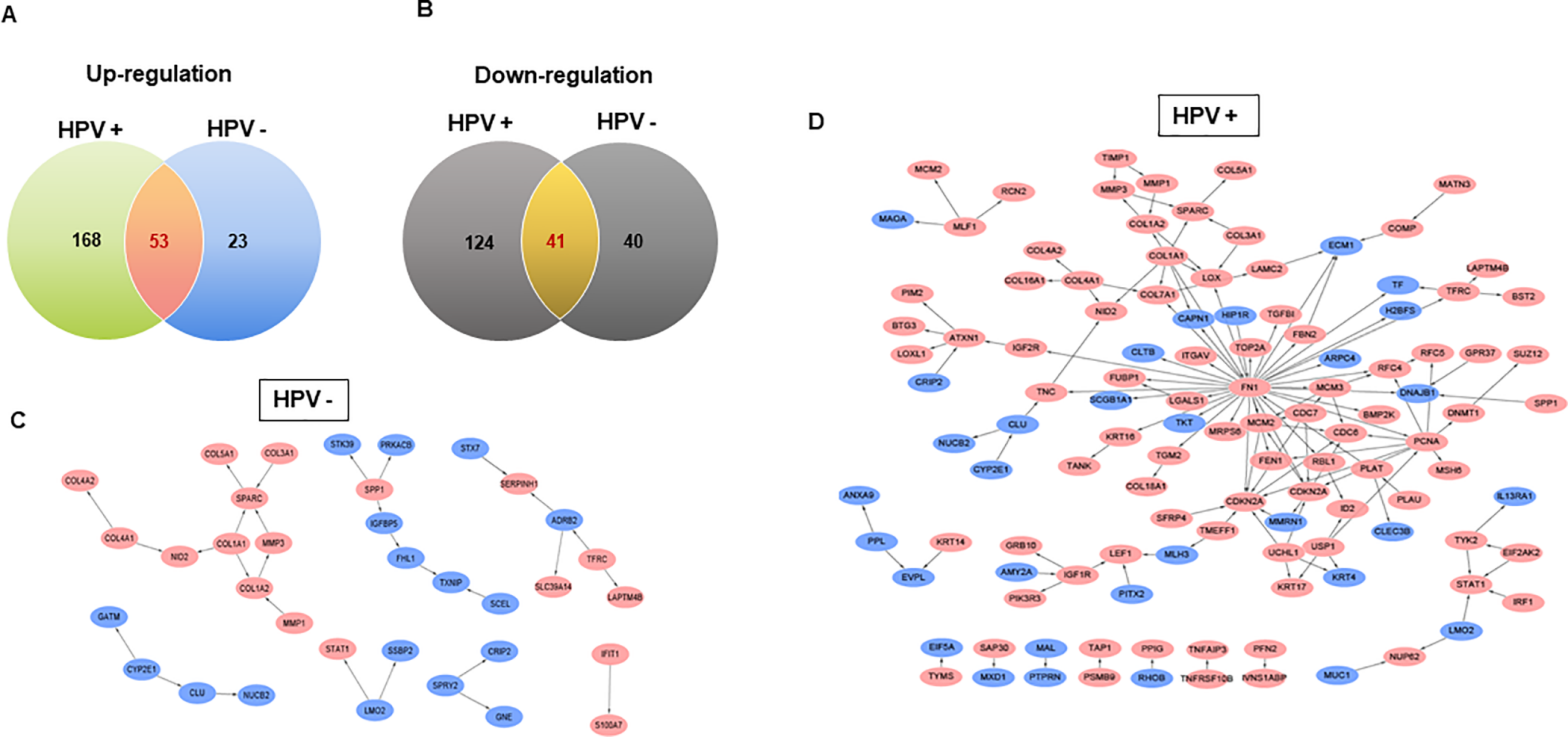
Network prediction from the differential gene expression patterns between HPV-positive and HPV-negative oral and oropharyngeal carcinoma. Venn diagram to illustrate the distinct gene expression between HPV-negative and HPV-positive head and neck cancer for (A) upregulated genes (B) downregulated genes. The number indicates the number of counted genes. (C) Gene expression networks of HPV-negative oral and oropharyngeal cancer patients. (D) Gene expression networks of HPV-positive oral and oropharyngeal cancer patients. Red: upregulated genes; blue: downregulated genes.

We assumed that HPV-positive oral and oropharyngeal cancers would be completely different from HPV-negative oral and oropharyngeal cancers as they have disparate natural sources. For a more fundamental analysis of the cancers, DEGs were collected from the publicly available data of Martinez et al., in which the authors provided the DEGs between HPV-negative and -positive oropharyngeal cancer. We re-analyzed the data and applied our own standards. Only genes with a fold-change or less than factor 2 and p-value below 0.05 were selected. These were expressed in a Venn diagram of the up- and downregulated genes (Fig 1A and 1B). Although genes common to both HPV-negative and positive oropharyngeal cancer were found, many genes were only found in one case. And the gene expression was compared with the data from The Cancer Genome Atlas (TCGA). We separately collected HPV negative (n = 415) and positive (n = 72) head and cancer samples. The common differentially expressed gene list was analyzed (S1 Fig). The protein interaction networks were identified with Cytoscape 2.0 and revealed that each cancer has a distinct signaling pathway and survival strategy. HPV-positive oral and oropharyngeal cancers triggered by viral infection showed a more complex gene profile in comparison with HPV-negative oral and oropharyngeal cancers (Fig 1C and 1D).

### E6 and E7 increase the expression of thymidylate synthase and topoisomerase II

**Fig 2:**
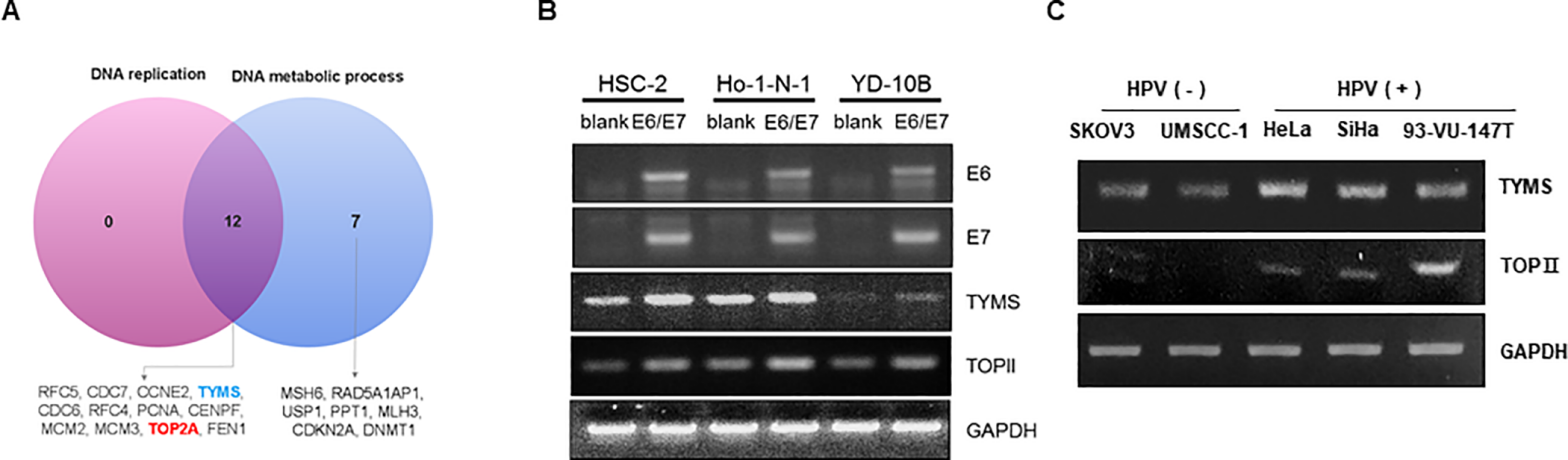
Association between HPV E6/E6 and TS and Topo II in oral cancer cell lines. (A) The classification of overexpressed genes in oral cancer, based on DAVID gene ontology analysis. (B) The expression changes in TS and Topo II after the transfection of E6/E6 genes into oral cancer cell lines. (C) Comparison of TS and Topo II expression between HPV negative and positive cell lines.

According to Fig 1, unlike HPV-negative head and neck cancer, several genes were overexpressed in HPV-positive head and neck cancer. In particular, pathways associated with DNA replication and DNA metabolic processes were overexpressed (Fig 2A). Among these genes, thymidylate synthase (TS) and topoisomerase II (Topo II) were commonly expressed in both pathways and are well-known druggable targets. To verify the validity of our experimental design for HPV status, we compared the genetic expression of HPV-negative oral cancer cells transfected with Lenti-blank (HPV-negative model) and Lenti-E6/7 (HPV-positive model). HPV E6 and E7 oncogenes, which play critical roles in the tumoral transformation of the host cell, are responsible for the onset and maintenance of head and neck cancer.[11, 22] In all cell lines, the enhanced expression of TS and Topo II was observed after transfection of Lenti-E6/E7 (Fig 2B). We collected three HPV- positive cell lines, Hela, SiHa and 93-VU-147T and compared the mRNA level of TS and Topo II gene with that of two HPV-negative cell lines SKOV3 and UMSCC-1. The HPV positive cells generally showed higher expression pattern (Fig 2C).

### Cisplatin is more effective in HPV-negative cells

**Fig 3:**
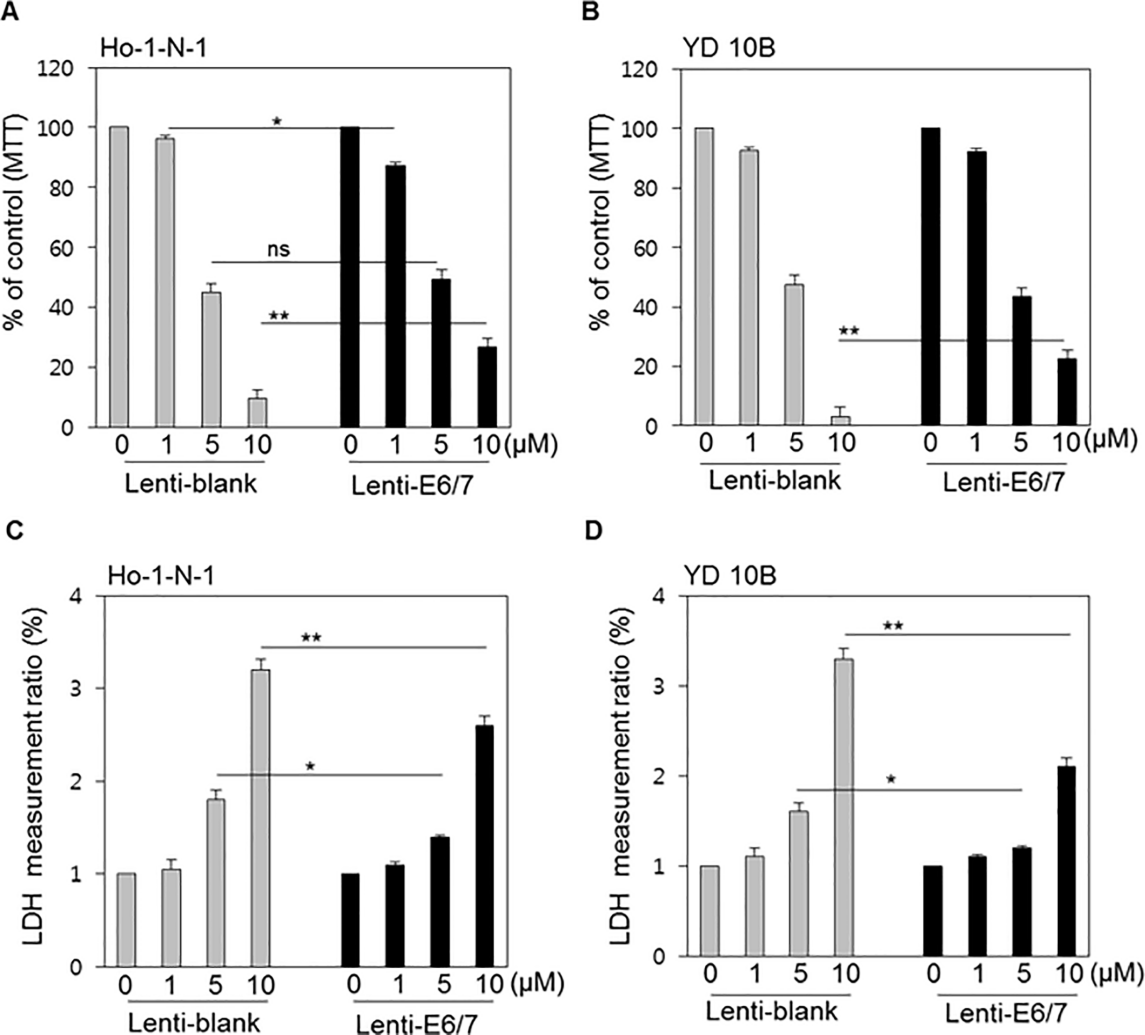
Cytotoxicity of cisplatin according to HPV status in oral cancer cell lines. (A, B) MTT assay results in Ho-1-N-1 and YD10B cells treated with 0, 1, 5, and 10 μM cisplatin after the transfection of Lenti-blank or Lenti-E6/7. The MTT assay indicates the cell viability. (C, D) LDH assay results for Ho-1-N-1 and YD10B cells, representative of HPV-negative and HPV-positive cell lines, respectively, were treated with increasing concentrations of cisplatin. Cell death was determined by LDH measurement. *p* values: * <0.05, ** <0.01.

To examine the differences in the cytotoxicities of chemotherapeutic drugs between HPV-positive cell lines and HPV-negative cell lines, the MTT assay was used to assess cell viability and the LDH assay to estimate cell death. Two oral cancer cell lines (Ho-1-N-1 and YD10B) were treated with cisplatin, which is used as the mainstay of head and neck cancer chemotherapy and concurrent chemoradiotherapy (CCRTx), and examined for any differences in response by HPV status.[23] In every experiment, cisplatin exerted a considerable anticancer effect in both HPV- and HPV+ models, in a dose-dependent manner (Fig 3). However, at higher concentrations, especially at 10 μM, highly significant differences between HPV-negative and HPV-positive models were observed. Cisplatin was more effective in the HPV-negative model cell line compared with HPV-positive model cell line.

### 5-FU works more effectively in HPV-positive cells

**Fig 4:**
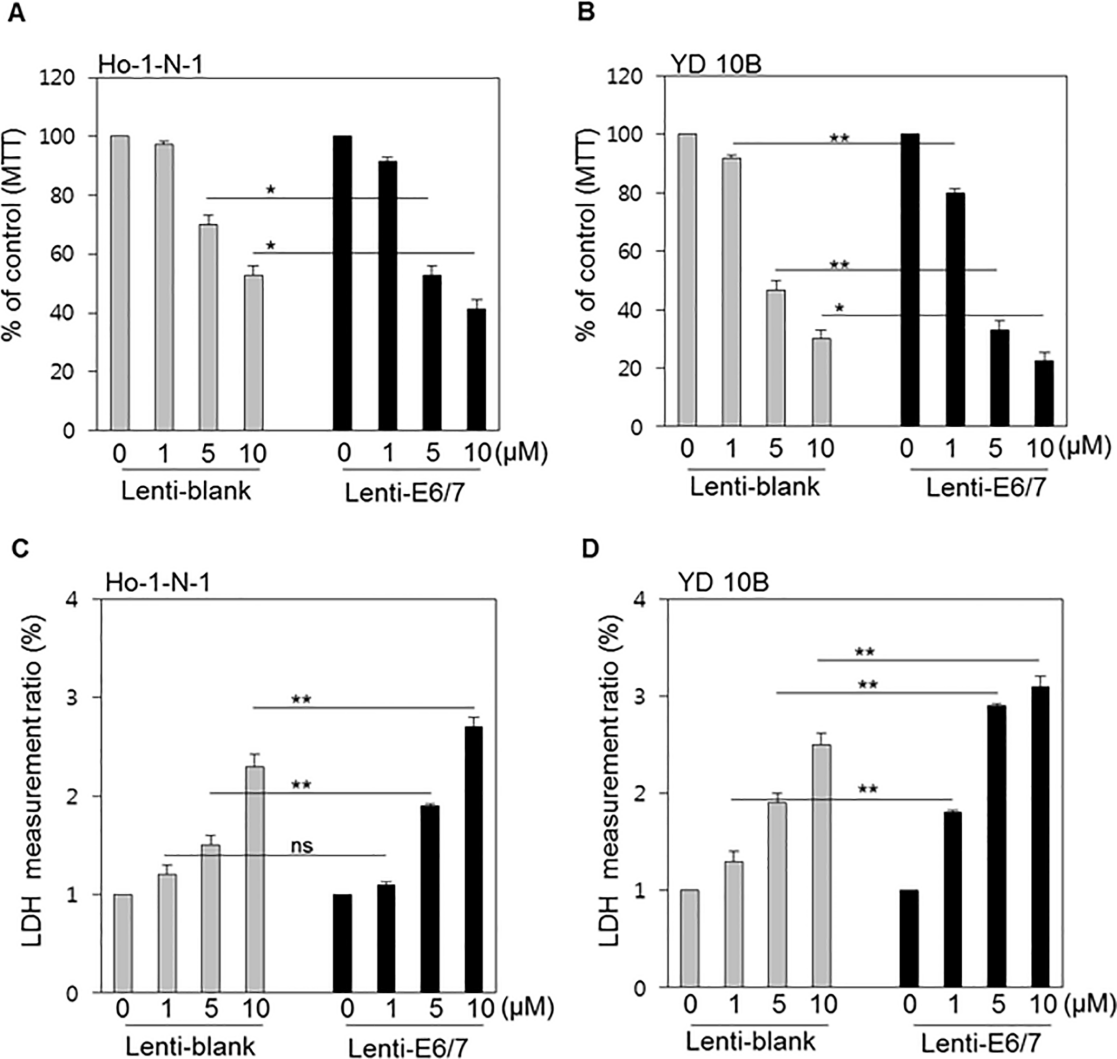
Cytotoxicity of 5-FU according to HPV status in oral cancer cell lines. (A, B) MTT assay results in Ho-1-N-1 and YD10B cells treated with 0, 1, 5, and 10 μM 5-FU after the transfection of Lenti-blank or Lenti-E6/7. (C, D) LDH assay results for Ho-1-N-1 and YD10B cells, representative of HPV-negative and HPV-positive cell lines, respectively, were treated with increasing concentrations of 5-FU. *p* values: * <0.05, ** <0.01.

Ho-1-N-1 and YD10B were treated with 5-FU, which is a TS inhibitor and used with cisplatin at CCRTx or in a palliative setting, depending on the concentrations. The use of 5-FU decreased oral cancer cell viability, regardless of the cell line or HPV status.[24] Although the effects of 1 μM 5-FU in Ho-1-N-1 cells were not different by HPV status, the cell viability of HPV-positive cell lines was more significantly diminished by 5-FU in both cell lines in comparison with the HPV-negative cell lines (Fig 4).

### Pemetrexed works more effectively in HPV-positive cells

**Fig 5:**
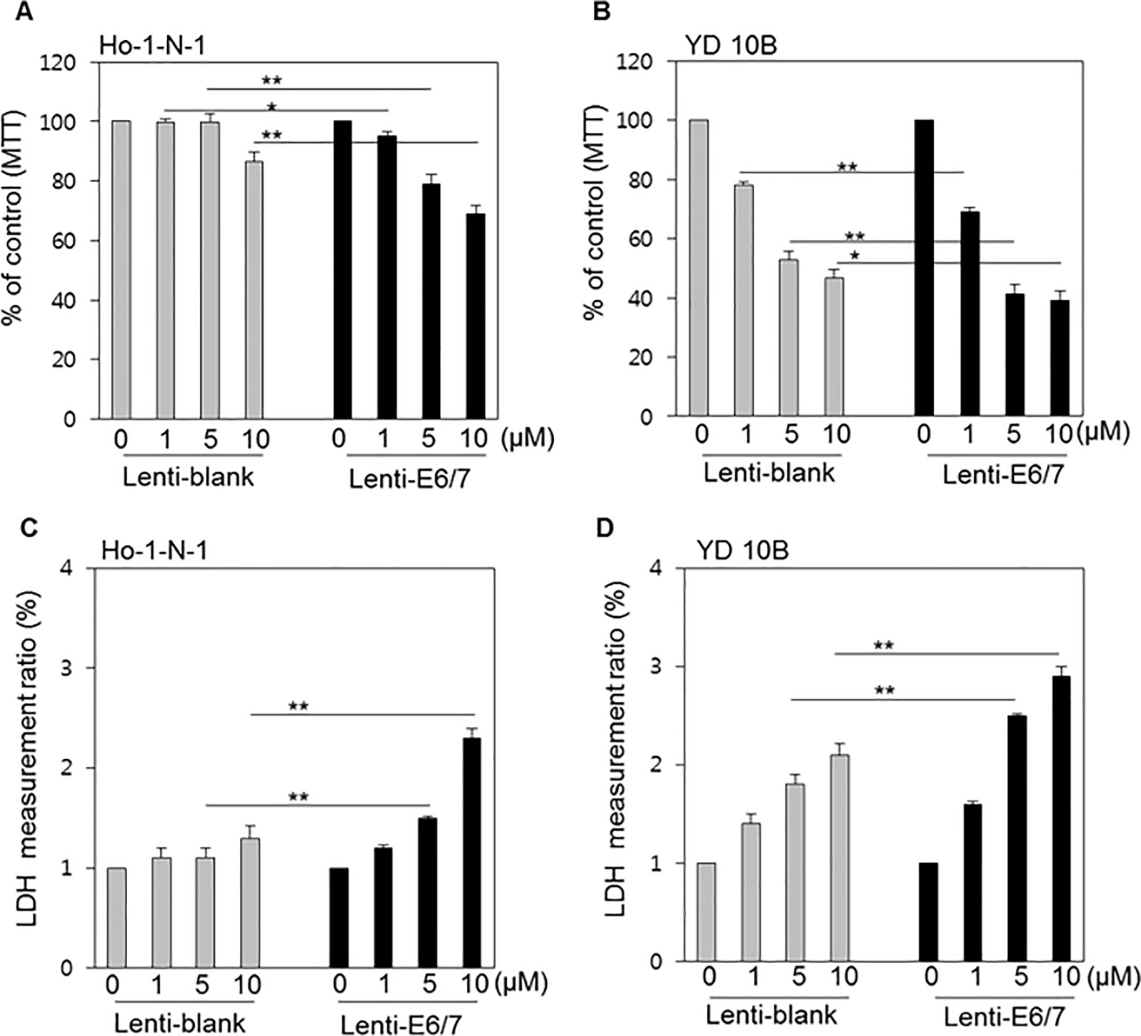
Cytotoxicity of pemetrexed according to HPV status in oral cancer cell lines. (A, B) MTT assay results in Ho-1-N-1 and YD10B cells treated with 0, 1, 5, and 10 μM pemetrexed after the transfection of Lenti-blank or Lenti-E6/7. (C, D) LDH assay result in Ho-1-N-1 and YD10B cells, representative of HPV-negative and HPV-positive cell lines, respectively, were treated with increasing concentrations of pemetrexed. *p* values: * <0.05, ** <0.01.

Ho-1-N-1 and YD10B were treated with pemetrexed, which is another TS inhibitor, in the range from 0–10 μM.[25] The MTT assay indicated that the cell viability of Ho-1-N-1 cells decreased gradually in a dose-dependent manner, whereas YD10B cells experienced a relatively sharper decline than Ho-1-N-1 cells (Fig 5A and 5B). The LDH assay revealed similar results in both cell lines (Fig 5C and 5D). More importantly, compared with the values obtained in equivalent conditions, HPV-positive cell lines were more sensitive in every experiment.

### Etoposide works more effectively in HPV-positive cells

**Fig 6:**
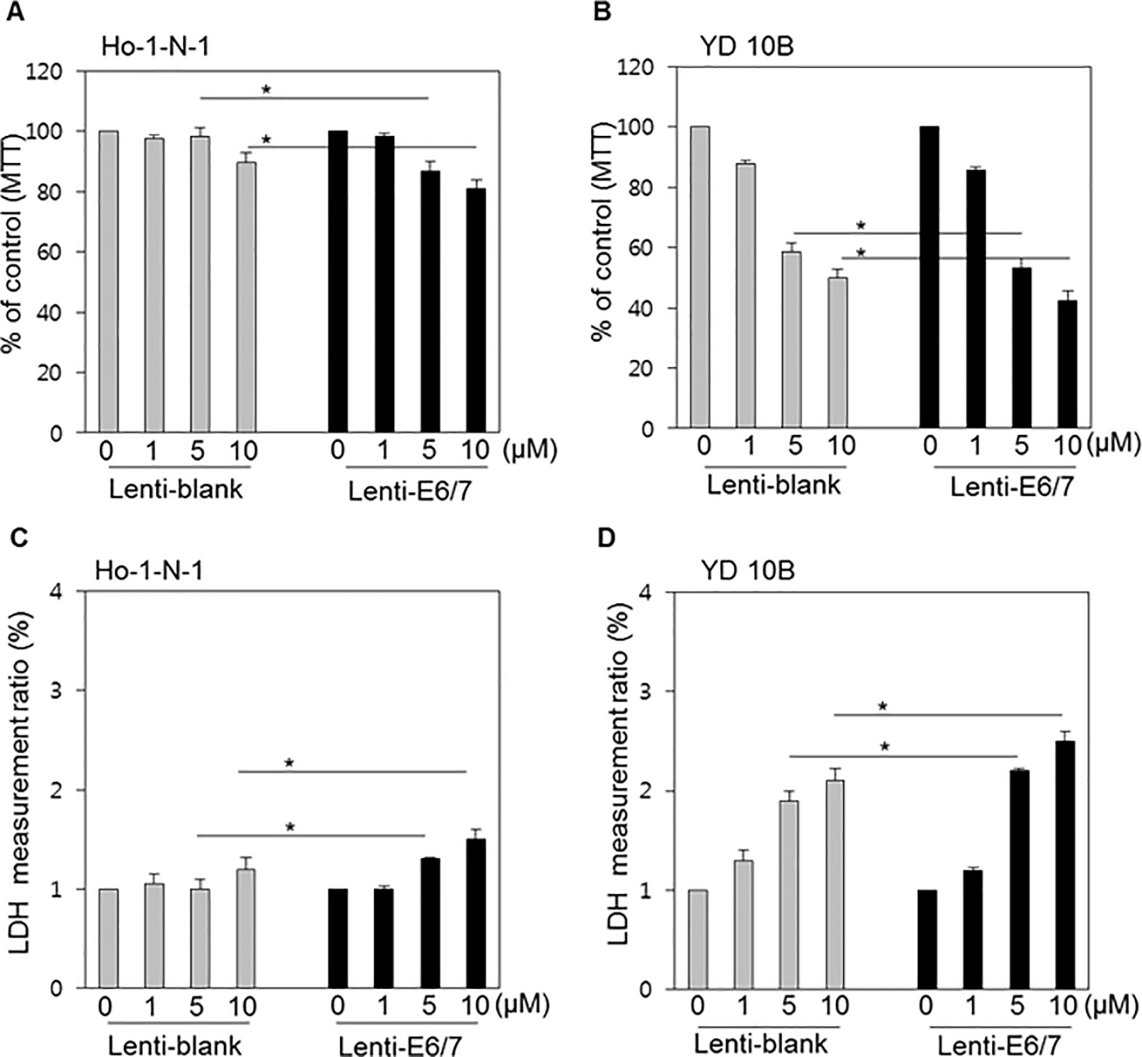
Cytotoxicity of etoposide according to HPV status in oral cancer cell lines. (A, B) MTT assay results in Ho-1-N-1 and YD10B cells treated with 0, 1, 5, and 10 μM of etoposide after the transfection of Lenti-blank or Lenti-E6/7. (C, D) LDH assay result in Ho-1-N-1 and YD10B cells, representative of HPV negative and HPV positive cell lines, were treated with increasing concentrations of etoposide. *p* values: * <0.05.

HPV-positive and HPV-negative oral cancer cells were treated with etoposide, a Topo II inhibitor, at different concentrations.[26] Etoposide is an anticancer agent and inhibits the growth of cancer cells in oral cancers regardless of cell line or HPV status (Fig 6). In addition, HPV-positive cells were more sensitive to the cell viability and cell death changes induced by etoposide; however, from a cell line perspective, YD10B cells reacted to etoposide better than Ho-1-N-1 cells.

### Cisplatin/etoposide and cisplatin/pemetrexed combination regimens showed synergic effects in HPV-positive cell lines

**Fig 7:**
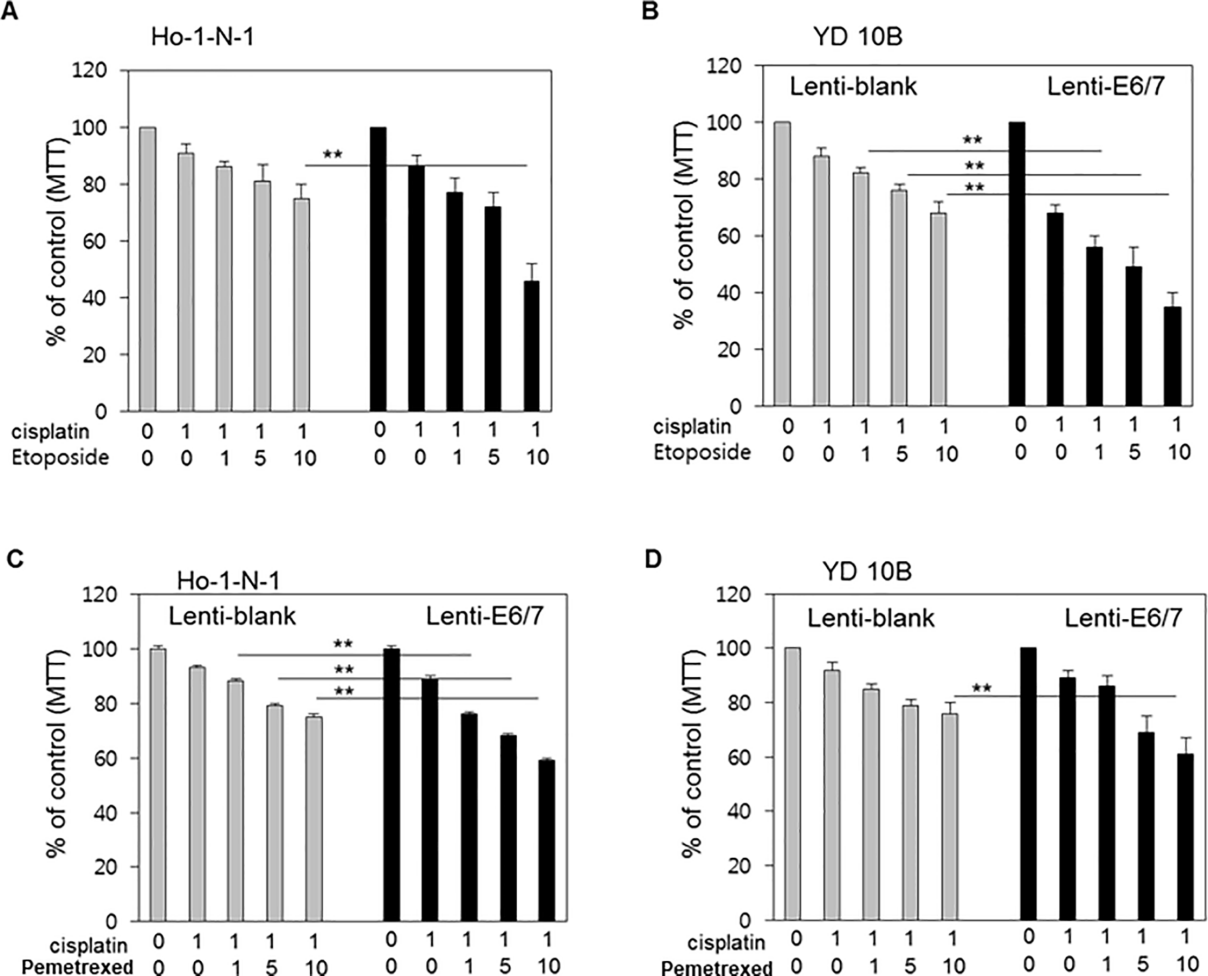
Cytotoxicity of cisplatin/etoposide or cisplatin/pemetrexed combination regimens according to HPV status in oral cancer cell lines. (A, B) MTT assay results in Ho-1-N-1 and YD10B cells treated with 1 μM cisplatin and 0, 1, 5, and 10 μM etoposide, according to HPV status. (C, D) MTT assay results in Ho-1-N-1 and YD10B cells treated with 1 μM cisplatin and 0, 1, 5, and 10 μM pemetrexed, according to HPV status. *p* values: ** <0.01.

**Fig 8:**
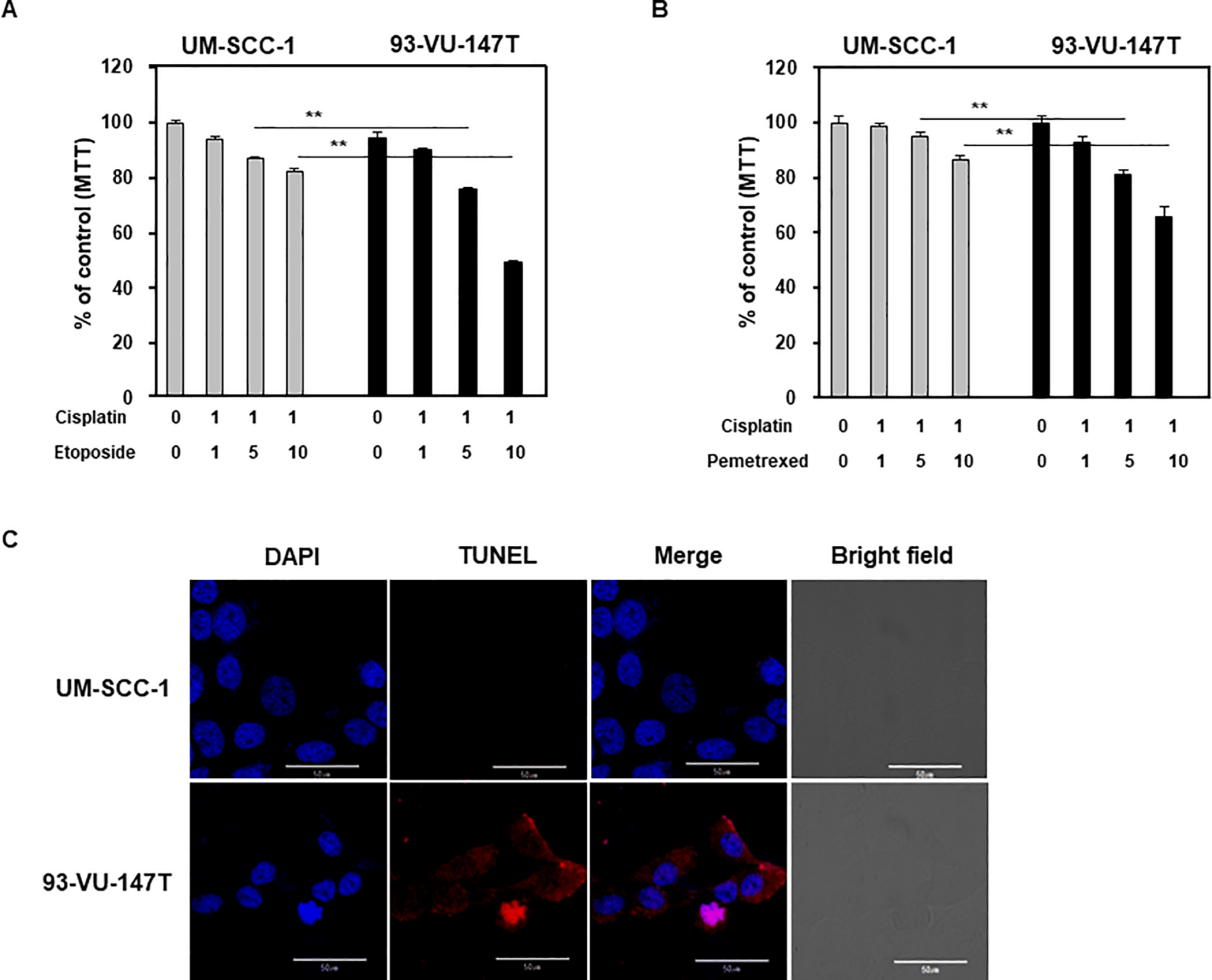
Cisplatin/etoposide or cisplatin/pemetrexed combination is more effectively in HPV positive HNSCC cell lines. (A) MTT assay results in UN-SCC-1 and 93-VU-147T cells treated with 1 μM cisplatin and 1, 5, and 10 μM etoposide. (B) MTT assay results in UN-SCC-1 and 93-VU-147T cells treated with 1 μM cisplatin and 1, 5, and 10 μM pemetrexed. (C) TUNEL assay results in UN-SCC-1 and 93-VU-147T cells treated with 1 μM cisplatin and 10 μM pemetrexed. *p* values: ** <0.01.

Low dose (1 μM) cisplatin was administered with various concentration of etoposide or pemetrexed in HPV-positive or -negative oral cancer cell lines. Although each combination regimen exerted gradual cytotoxicity in a dose-dependent manner, the effects were more prominent in HPV-positive oral cancer cell lines (Fig 7). The cytotoxicity of the cisplatin/etoposide combination regimen was more prominent in HPV-positive YD10B cell lines (Fig 7A and 7B). In contrast, the cytotoxicity of the cisplatin/pemetrexed combination regimen was more prominent in HPV-positive Ho-1-N-1 cells (Fig 7C and 7D). Next, to further study the effect of head and neck squamous cell carcinoma (HNSCC) cell lines, we used 93-VU-147T (HPV positive) and UM-SCC-1 (HPV negative) cell lines. Cisplatin/etoposide and cisplatin/pemetrexed combination treatment significantly decreased 93-VU-147T cell viability (Fig 8A and 8B), and cisplatin/pemetrexed treatment induced 93-VU-147T cell death (Fig 8C). These result shows that transfected oral cancer cell and HNSCC cell lines were observed similar effect in each drug.

## Discussion

In this article, we revealed the differences in the gene expression patterns between HPV-negative head and neck cancer and HPV-positive head and neck cancer and identified druggable targets, such as TS and Topo II, in HPV-positive head and neck cancer from big data derived patients with head and neck cancer patients. To mimic HPV infection in HPV naïve oral and oropharyngeal cancer cells, the transfection of E6 and E7, which are oncogenes of HPV, was performed. As shown in the bio-informatics data, the insertion of E6 and E7 increased the expression of TS and Topo II, which contributed to the development and progression of cancer. Although the HPV-negative oral cancer cell lines are more susceptible to cisplatin, which is the mainstay of previously established chemotherapy regimens of head and neck cancer, the inhibition of TS by 5-FU or pemetrexed and the inhibition of Topo II by etoposide exerted greater cytotoxicity in HPV-positive oral and oropharyngeal cancer cell lines in a dose-dependent manner. Furthermore, the addition of pemetrexed and etoposide to cisplatin showed synergistic effects on HPV-positive oral and oropharyngeal cancer cell lines.

As previously mentioned, because there are insufficient data to justify a change the treatment modalities based on upon HPV status, patients with head and neck cancer currently receive the same treatment modalities and chemotherapeutic regimens, regardless of their HPV status.[16] Although cetuximab and immune checkpoint inhibitors, such as nivolumab and pembrolizumab, have been recently introduced in head and neck cancer treatment, cisplatin-based chemotherapy or concurrent chemoradiotherapy are still the preferred regimens in locally advanced or metastatic head and neck cancer patients.[15, 16, 23, 27, 28]

In previous study, Ijuin et al studied correlation between prognosis of advanced oropharyngeal cancer patients and TS or thymidylate phosphorylase.[29] However, they did not conduct the experiments on the presence of HPV. Similar to non-small cell lung cancer, pemetrexed was also previously used with cisplatin as a first-line treatment in recurrent or metastatic head and neck cancer.[30, 31] Unfortunately, the cisplatin/pemetrexed combination regimen did not improve objective response rate (RR; 48% vs 32%, respectively) and overall survival (OS; 7.3 months vs. 6.3 months, respectively) compared with a cisplatin/placebo regimen group in a double-blind, randomized phase II trial.[31] However, the cisplatin/pemetrexed combination regimen showed an improvement of OS and progression-free survival (PFS) for patients with oropharyngeal cancer in subgroup analysis (hazard ratio: 0.59). Although a difference in survival was not shown in the overall group of mixed HPV-positive and -negative patients, it is thought that OS and PFS improved in the oropharyngeal cancer subgroup because the subgroup included many HPV-positive patients. Similarly, carboplatin/pemetrexed combination regimen showed an improvement of PFS (7.0 months) and OS (17.1 months) in HPV positive oropharyngeal cancer patients subgroup compared to PFS (5.1 months) and OS (9.4 months) in overall head and neck cancer patients.[32] This result was further strong evidence that supported our hypothesis. Gemcitabine/pemetrexed also proved to be an effective (PR: 36%) and safe combination for advanced head and neck cancer, which results in a large number (38%) of oropharyngeal cancer patients.[33] Pemetrexed was also used alone or with other target agents, such as bevacizumab or cetuximab, in head and neck cancer. Pemetrexed alone showed moderate activity (RR: 26.5%, disease control rate (DCR): 70.6%) for patients with recurrent locally advanced or metastatic head and neck cancer and had manageable toxicities.[34] Because this article did not categorize the location of head and neck cancers, it was difficult to evaluate the response to pemetrexed according to HPV status. In another study, bevacizumab was administered with pemetrexed to potentiate the activity of pemetrexed and the pemetrexed/bevacizumab combination regimen showed promising efficacy (RR: 30% including 5% complete remission (CR), DCR: 87%) in recurrent or metastatic squamous head and neck cancers.[35] As 50% of the enrolled patients comprised patients with oropharyngeal cancer, who have a higher HPV infection rate, a positive result was expected. Pemetrexed/cisplatin/cetuximab regimen also showed promising results (RR: 29.3%, DCR: 69%).[36] As is well known, pemetrexed combined regimens did not show significant toxicities in these studies.

Etoposide was also previously used for the treatment of patients with head and neck cancers. Although oral etoposide showed dramatic efficacy (RR: 91% including 82% CR and partial response (PR): 9%) with radiation therapy in a study with a large percentage of patients with oropharyngeal cancer (50%), oral etoposide showed modest efficacy (RR: 8%, DCR: 43%) in a study with a smaller percentage of patients with oropharyngeal cancer (11%).[37, 38] Although there were differences in the clinical settings of the two studies (chemoradiation vs palliative setting), oral etoposide showed more efficacy in studies with a larger proportion of patients with higher HPV prevalence, without causing any significant toxicity. Although significant myelosuppression occurred, an etoposide/cisplatin/bleomycin combination regimen also resulted in a good response (RR: 70% including CR: 7%) in recurrent and metastatic head and neck cancer.[39]

## Conclusions

We identified why different approaches should be used, depending on HPV status, and identified TS and Topo II as druggable targets for HPV-positive oral and oropharyngeal cancer using “big data” derived from patients with head and neck cancer and bio-informatics techniques. In addition, we demonstrated differences in the efficacy depending on HPV status in model HPV oral and oropharyngeal cancer cell lines. We suggested that pemetrexed or etoposide in combination with cisplatin, can be used in a regimen of definitive concurrent chemoradiotherapy instead of cisplatin alone regimen for HPV-positive oral and oropharyngeal cancer patients. In addition, in case the cancer progresses despite the use of immunotherapy, pemetrexed or etoposide can be used a palliative regimen for recurrent or metastatic HPV-positive oral and oropharyngeal cancer patients. Although HPV prevalence in other head and neck cancers is lower than that in oropharyngeal cancer, if presence of HPV is confirmed, pemetrexed or topotecan need to be considered as another treatment option. However, a well-designed clinical trial is needed to test this in patients with HPV-positive oral and oropharyngeal cancer.

## Supporting information

S1 Fig Common differentially expressed gene list between Martinez et al. and TCGA on HPV- positive head and neck squamous cancer. (A) up-regulated gene list (B) Down-regulated gene list. Thymidylate synthase (TS) and topoisomerase II (Topo II) are highlighted.
(TIF)
