# Supplementary figures and images for: Evaluation of pemetrexed and etoposide as therapeutic regimens for human papillomavirus-positive oral and oropharyngeal cancer
Source: PLoS One. 2018 Jul 11;13(7):e0200509. doi: 10.1371/journal.pone.0200509 (PMC6040768; doi:10.1371/journal.pone.0200509)

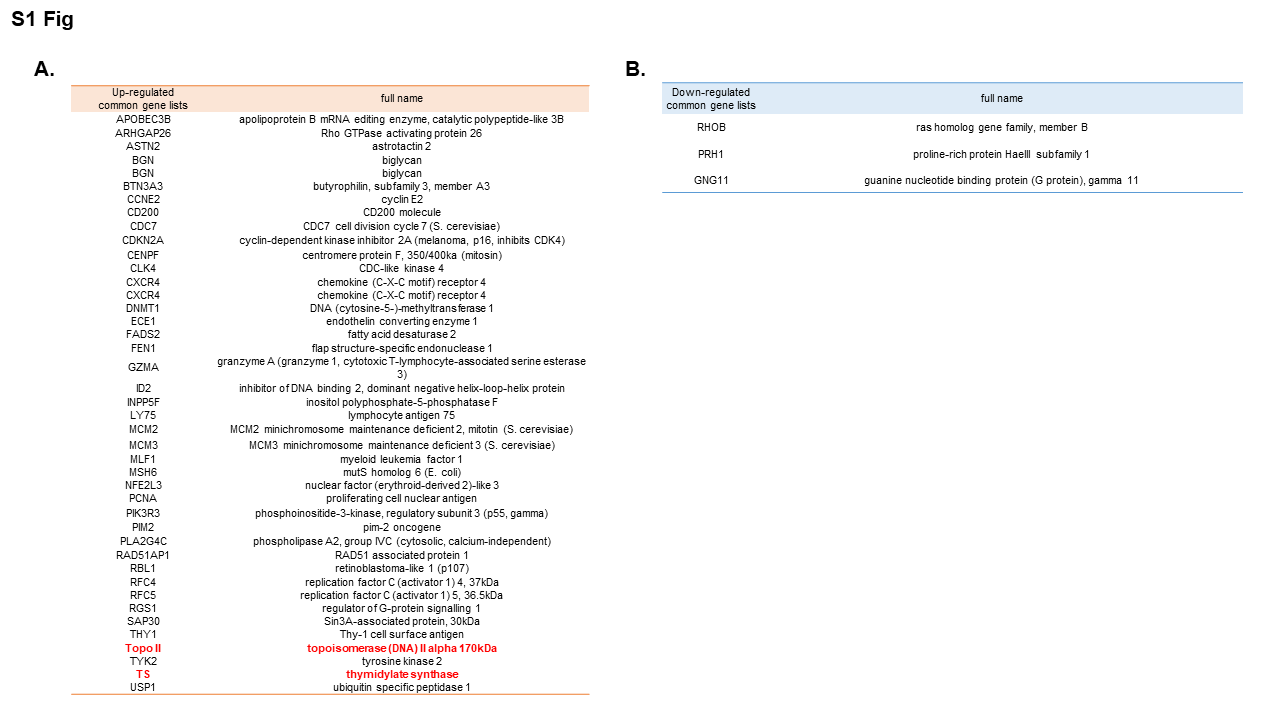

Supplement: S1 Fig — (A) up-regulated gene list (B) Down-regulated gene list. Thymidylate synthase (TS) and topoisomerase II (Topo II) are highlighted. (TIF) [file pone.0200509.s001.tif]
